# Ensemble approach combining multiple methods improves human transcription start site prediction

**DOI:** 10.1186/1471-2164-11-677

**Published:** 2010-11-30

**Authors:** David G Dineen, Markus Schröder, Desmond G Higgins, Pádraig Cunningham

**Affiliations:** 1 Complex and Adaptive Systems Laboratory (CASL), University College Dublin, Belfield, Dublin 4, Ireland; 2 The Conway Institute of Biomolecular and Biomedical Research, University College Dublin, Belfield, Dublin 4, Ireland; 3 BRF, Center for Biotechnology (CeBiTec), Bielefeld University, Bielefeld, Germany

## Abstract

**Background:**

The computational prediction of transcription start sites is an important unsolved problem. Some recent progress has been made, but many promoters, particularly those not associated with CpG islands, are still difficult to locate using current methods. These methods use different features and training sets, along with a variety of machine learning techniques and result in different prediction sets.

**Results:**

We demonstrate the heterogeneity of current prediction sets, and take advantage of this heterogeneity to construct a two-level classifier ('Profisi Ensemble') using predictions from 7 programs, along with 2 other data sources. Support vector machines using 'full' and 'reduced' data sets are combined in an either/or approach. We achieve a 14% increase in performance over the current state-of-the-art, as benchmarked by a third-party tool.

**Conclusions:**

Supervised learning methods are a useful way to combine predictions from diverse sources.

## Background

The field of in-silico promoter prediction has developed greatly in recent years. Machine learning techniques, such as support vector machines and self-organising maps, and new features, especially those associated with structural properties of the DNA molecule, have led to progressive improvements in accuracy. The realization that the majority of the genome is transcribed [1-3], and that most promoters have diffuse clusters of multiple transcription start sites (TSS) [4], has led to a move away from discrete predictions, and towards scores for all base pairs of the genome. There is greater consensus on the correct way to evaluate predictions, reducing the biases inherent in the plethora of methods previously used [5].

**Figure 1:**
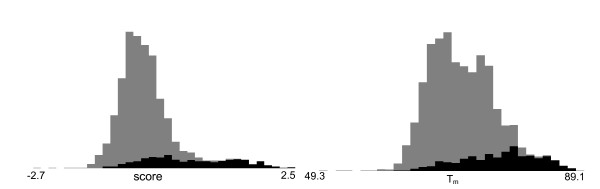
**Promoter histogram scores**. Histograms of scores from (a) ARTS, and (b) Profisi on 519 dbTSS promoters and 2,595 non-promoters drawn from the ENCODE regions. ARTS scores are the output of an SVM, while Profisi scores are DNA melting temperatures. Promoters are black and non-promoters are grey. Specificity is high at high thresholds, but many promoters are given low scores, and are obscured by non-promoters.

Despite these developments, there is considerable need for improvement in promoter prediction performance. A bimodal distribution of CpG content splits human promoters into high and low-CpG content promoters [6]. Promoters with lower CpG content are associated with tissue-specific regulation [7], and are considered more difficult to predict [8]. Figure 1 shows histograms of scores for ARTS [9] and Profisi [10], two state-of-the-art methods. Many valid promoters receive low scores, and setting thresholds low enough to recover them will inevitably return many false positives.

One obvious way of improving performance is to combine several existing methods using an ensemble learning approach. Ensembles combining results from multiple programs have seen some use in promoter prediction [11,12]. They have also been successfully used in several other computational biology problem areas [13-15]. High diversity in individual methods is considered predictive of good ensemble accuracy [16]. It can be difficult, however, to improve on the performance of the best individual method [17]. In this paper, our aim was to explore whether the set of prediction methods was indeed diverse, and to improve predictive performance across the genome and at all thresholds.

Table 1 shows our chosen features. Most of the features are programs which were drawn from the top performers in a number of promoter prediction reviews [5,18]. It includes our own Profisi method [10], which we have previously shown to be very competitive. These programs are diverse in both features used and in machine learning methods. In addition, we included methylation profiles [19] and conservation scores from phylogenetic comparisons. Profisi is based on the observation that promoters are associated with high DNA melting temperature. DNA methylation both lowers melting temperature [20] and blocks promoter activity [21], but is not accounted for in the model used by Profisi. Hence, including methylation data could boost Profisi's performance. High conservation scores are considered predictive of functional areas of DNA [22].

**Table 1:** Description of features used.

Feature	Information
Profisi	DNA melting temperature, as calculated with Fixman & Freire's method
ARTS	Custom SVM kernel using both sequence and structural information
N-SCAN	HMM gene predictor - start of 5' UTR defines TSS
FirstEF	Decision tree using *k*-mers, GC and CpG content
Eponine	RVM using mixture of Gaussian distributions of position weight matrices
ProSOM	Self-organising map trained on base stacking energy
EP3	Base stacking energy
Methylation	Experimentally determined CpG methylation profiles
Conservation	17-way vertebrate conservation scores

All except methylation and conservation are outputs from prediction programs. ARTS scores were split into + and - strands. Methylation scores were split into stem cell and differentiated categories.

As not all of our features are prediction scores, we did not use an averaging or voting-based ensemble method. Instead we used a support vector machine for aggregation. This also gave us the opportunity to use a non-linear kernel to increase separability of promoter and non-promoter classes.

MetaProm [11] and EnsemPro [12] are both programs that use ensemble methods for promoter prediction. Although we were unable to obtain predictions for these programs, we could evaluate Profisi Ensemble using the evaluation rules described in the original papers in an attempt to make some comparison with them. MetaProm is based on an artificial neural network, and makes predictions in an area covering around 30% of the human genome, using a combination of dbTSS and RefSeq as the reference set. Multiple methods are discussed in the EnsemPro paper, but the most successful is weighted majority voting. It restricts its predictions to an area 1,150 base pairs either side of 400 TSS drawn from the Eukaryotic Promoter Database (EPD). The EPD is known to be strongly biased towards TATA box-containing promoters, which only comprise a small fraction of human promoters as a whole [23].

## Results and Discussion

Table 2 shows the overlap between sets of predictions from seven popular promoter prediction programs, based on whole genome predictions for assembly hg17 of the human genome. Both true and false positives were counted, as variation in both will improve ensemble performance. Predictions from only two pairs of programs overlapped by 50% or more. The highest overlap was between ProSOM [24] and EP3 [25], which are by the same authors and use the same features. FirstEF [26], Eponine [27], and N-SCAN [28] also had reasonable overlap between prediction sets. The average overlap between pairs of predictions was 31.6%. The low average overlap suggested that an ensemble approach was worth pursuing, as the ensemble would have good diversity. We further analysed overlap by splitting predictions into true (Table 3) and false (Table 4) positives. There was more overlap between true predictions than between false predictions (54% versus 21%). In other words, different programs differed more in the mistakes they made than in their correct predictions.

**Table 2:** Overlap between whole genome predictions as measured by ∩/∪ (1000 bp tolerance).

**N-SCAN**	1.000						
**FirstEF**	0.424	1.000					
**Eponine**	0.442	0.440	1.000				
**ProSOM**	0.212	0.284	0.256	1.000			
**EP3**	0.209	0.317	0.244	0.575	1.000		
**ARTS**	0.308	0.314	0.319	0.161	0.162	1.000	
**Profisi**	0.319	0.504	0.360	0.255	0.290	0.247	1.000
	**N-SCAN**	**FirstEF**	**Eponine**	**ProSOM**	**EP3**	**ARTS**	**Profisi**

Where more than one prediction existed in a 2,000 bp range, only the prediction with the highest score was counted. For ProSOM, EP3, ARTS, and Profisi, predictions were thresholded to leave ~20,000 predictions per program.

**Table 3:** Overlap between whole genome predictions as measured by ∩/∪ (1000 bp tolerance), considering only true positive predictions.

**N-SCAN**	1.000						
**FirstEF**	0.740	1.000					
**Eponine**	0.569	0.624	1.000				
**ProSOM**	0.384	0.432	0.436	1.000			
**EP3**	0.413	0.473	0.457	0.704	1.000		
**ARTS**	0.626	0.688	0.612	0.401	0.428	1.000	
**Profisi**	0.579	0.661	0.580	0.414	0.445	0.581	1.000
	**N-SCAN**	**FirstEF**	**Eponine**	**ProSOM**	**EP3**	**ARTS**	**Profisi**

**Table 4:** Overlap between whole genome predictions as measured by ∩/∪ (1000 bp tolerance), considering only false positive predictions.

**N-SCAN**	1.000						
**FirstEF**	0.218	1.000					
**Eponine**	0.294	0.315	1.000				
**ProSOM**	0.109	0.210	0.164	1.000			
**EP3**	0.103	0.244	0.149	0.523	1.000		
**ARTS**	0.136	0.163	0.174	0.082	0.080	1.000	
**Profisi**	0.168	0.416	0.241	0.195	0.232	0.131	1.000
	**N-SCAN**	**FirstEF**	**Eponine**	**ProSOM**	**EP3**	**ARTS**	**Profisi**

**Figure 2:**
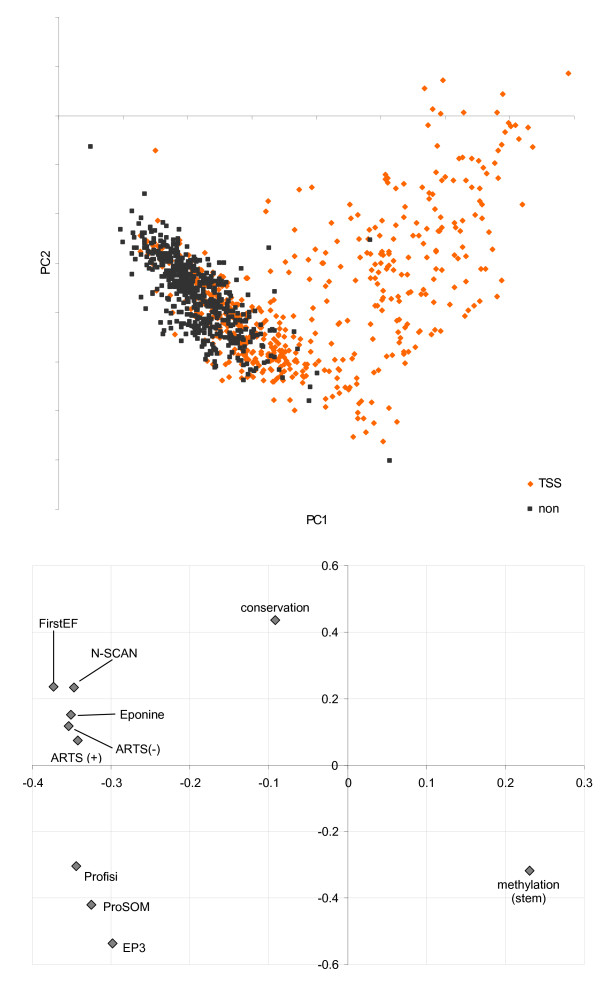
**Training set PCA plot**. (a) First two principal components of training set (519 dbTSS promoters and 2,595 non-promoters). (b) plot of PCA feature weights for first two principal components.

Principal components analysis (PCA) of the training set (519 promoters and 2,595 non-promoters) was used to give a rough visual representation of the separability of the promoter and non-promoter classes. The first two principal components are plotted in Figure 2a. No one feature was noticeably highly weighted in the first two principal components. Non-promoters form a reasonably tight cluster, while promoters are much more diffuse. This is a consequence of using promoter-centric features. Naively, it would be expected that the non-promoter class would be more diffuse, given the many different types of DNA it comprises. The feature weights from these principal components are plotted in Figure 2b. Promoter features appear together, in two groups, while conservation and methylation features are both separate.

Evaluating the contribution of each feature in a support vector machine can be difficult and computationally expensive. We therefore used information gain-based feature ranking to predict the potential contribution of each feature. The predicted ranking is shown in table 5. ARTS [9] was ranked top, which was not surprising given its status as best performer in the last comprehensive prediction review [5]. Surprisingly, the ranking of methylation scores was comparable to many supervised methods. This may be because we only had nonzero scores for CpG islands, areas associated with promoter activity in general. Conservation was the lowest ranked feature by some distance. This implies that it is only beneficial when combined with other features.

**Table 5:** Information gain-based ranking of features based on analysis of the training set with Weka 3.6 using default parameters.

Score	Feature
0.196	ARTS (+ strand)
0.195	ARTS (- strand)
0.182	Profisi
0.161	FirstEF
0.157	N-SCAN
0.153	Methylation (differentiated cells)
0.150	Methylation (stem cells)
0.113	Eponine
0.105	ProSOM
0.090	EP3
0.031	17-way conservation

The top 5 features in this ranking were used to train the 'high specificity' SVM.

**Figure 3:**
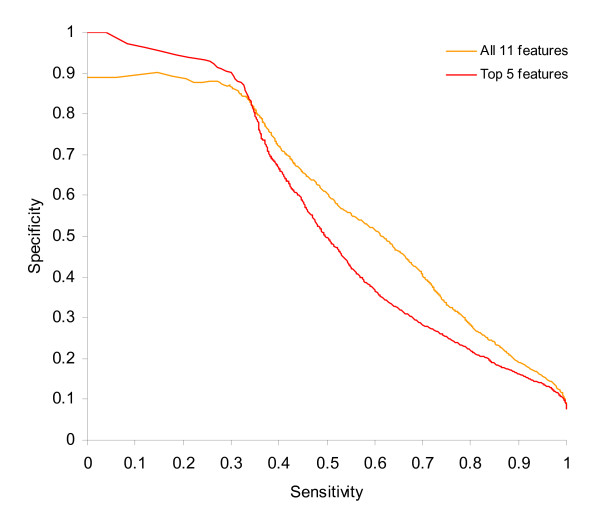
**Program output**. Tracks for Profisi Ensemble and its constituent features in a 7,500 bp area around the VPS72 promoter, as viewed in the UCSC Genome Browser. The output from Profisi Ensemble accurately predicts the promoter location. Eponine, N-SCAN, stem cell methylation, and differentiated cell methylation scores have been omitted as they contain no values in this area.

We trained SVMs using both the full set of 11 features, and a reduced set of the 5 top features ranked by information gain. Both were tested on human chromosome 22 only using pppBenchmark 1.3 [5]. The mapped area of chromosome 22 corresponds to ~1% of the genome. Sensitivity-specificity curves for these tests are shown in Figure 3. The reduced feature set is more accurate at high thresholds, while the full set is more accurate at low thresholds. This held true even when we tried a large range of different parameters for both C (error penalty) and γ (Gaussian width). It was decided to combine results from both models for the whole genome test. We used the reduced model unless its predicted probability fell below 0.94, in which case the full model was used instead. This idea of "punting" - switching classifiers when the score falls below a certain threshold - has been successfully used in protein family prediction [29]. To verify the soundness of the idea, we reran pppBenchmark with the combined predictions. The area under the resulting curve was the same as the area under the union of the two previous results. Having learned our threshold on 1% of the data, we then calculated predictions for the whole genome.

**Figure 4:**
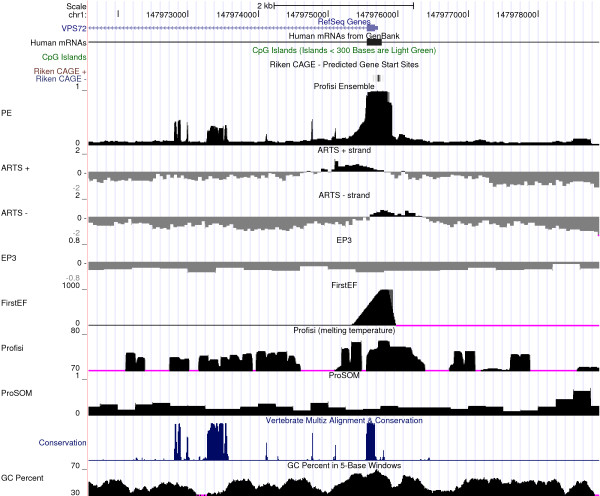
**Full versus reduced training sets**. Sensitivity-specificity curves for full feature set versus top five features selected via information gain, tested on chromosome 22 with protocol 2A (CAGE). The final classifier uses the top 5 features by default, but switches to all 11 features below the point at which the curves cross.

An example of one of the final predictions is given in Figure 4. Shown is the area 3 kbp around the VPS72 promoter, a promoter not associated with a CpG island. Plots of features with nonzero values in this area, as well as GC content, are shown beneath the main prediction. The peak in the Profisi Ensemble score coincides well with both the RefSeq and CAGE annotations. In addition, scores either side of the peak are very low, indicating the reduction in noise achieved by the ensemble approach, compared to individual predictors.

Whole genome predictions were evaluated using pppBenchmark 1.3. pppBenchmark evaluates predictions versus cap analysis of gene expression (CAGE) and RefSeq annotations, using both binning and distance-based protocols, for an accurate overall view of predictive power. The best performer in the original benchmarking was ARTS.

**Figure 5:**
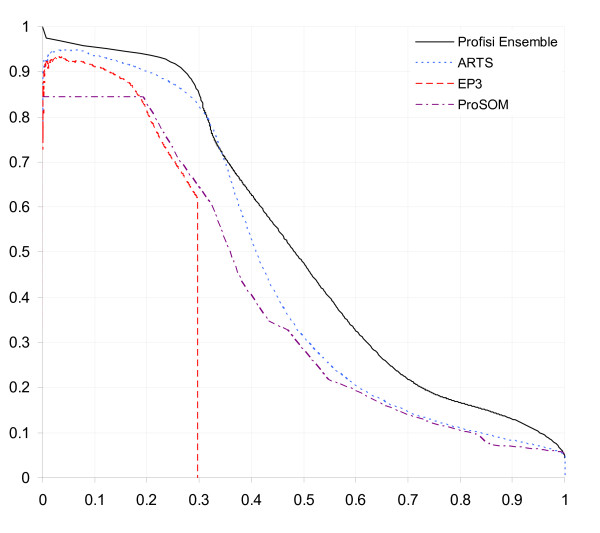
**Performance comparison (sensitivity-specificity scores)**. Sensitivity-specificity curves for various promoter prediction programs, tested on human genome build hg18 with protocol 2A (CAGE).

Figure 5 shows sensitivity-specificity curves for a number of programs using pppBenchmark's protocol 2A (considered the most important score by pppBenchmark's authors). Profisi Ensemble shows the best performance over the vast majority of the curve. Curves for CpG and non-CpG performance are given in Additional file 1.

**Figure 6:**
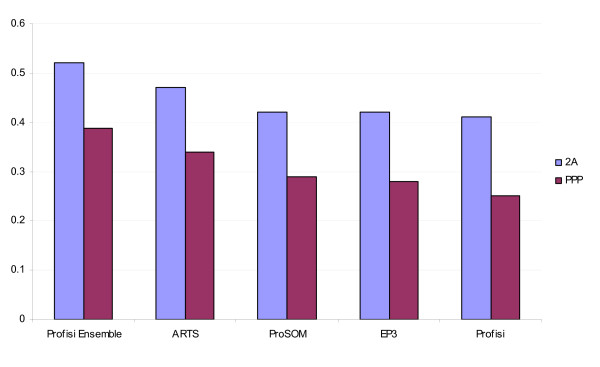
**Overall performance as evaluated by pppBenchmark**. Protocol 2A represents distance-based performance on CAGE tags, while PPP is the harmonic mean of four separate measurements using both CAGE and gene annotation, and represents overall predictive power. Scores for ARTS, ProSOM, and EP3 were taken from the original pppBenchmark evaluation.

Profisi Ensemble's pppBenchmark performance was as follows: 1A: 0.224 1B: 0.407 2A: 0.520 2B: 0.691. This is an improvement over ARTS in all categories. The PPP score is defined as the harmonic mean of the four scores given above. Profisi Ensemble's PPP score was 0.389, or 14% better than ARTS. Figure 6 shows these 2A and PPP scores. In summary, Profisi Ensemble is currently the most accurate predictor of human promoter activity.

We also performed comparisons with MetaProm and EnsemPro. As we did not have access to predictions from these programs, we evaluated Profisi Ensemble using their evaluation rules.

**Figure 7:**
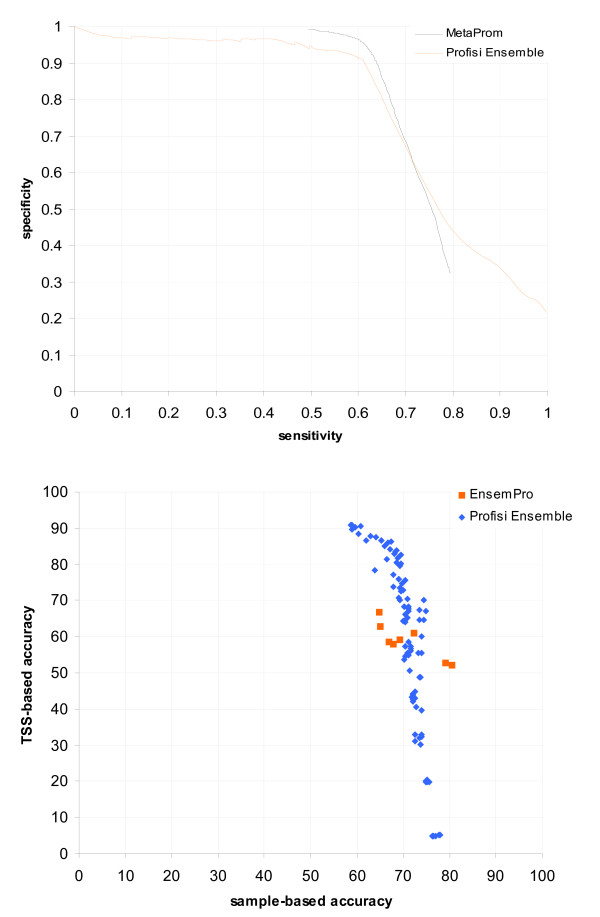
**Comparison with other ensemble methods**. (a) Evaluation of Profisi Ensemble and MetaProm, using 42,536 TSS drawn from dbTSS and refSeq, in the areas covered by the dbTSS annotation, with a tolerance of 2,000 bp. (b) Evaluation of Profisi Ensemble and EnsemPro (weighted average voting), in random 1.5 kbp areas around Eukaryotic Promoter Database start sites, with a tolerance of 200 bp upstream and 100 bp downstream. EnsemPro scores represent the average of multiple runs.

MetaProm uses a combination of dbTSS and RefSeq as its evaluation set, taking not only a single representative TSS from dbTSS, but also the most upstream and downstream, to evaluate performance in the prediction of alternative TSS. Predictions within 2 kbp of the TSS were considered valid. The results of our evaluation are shown in Figure 7a. MetaProm performed better at high specificities, while Profisi Ensemble performed better at high sensitivities.

EnsemPro uses the EPD as its reference set. As mentioned above, the EPD is not considered a representative set of human promoters. Only an area 1.5 kbp in size around the TSS was examined. Predictions within 200 bp (upstream) or 100 bp (downstream) were counted as true positives. The results of the evaluation are shown in Figure 7b. Profisi Ensemble shows roughly equivalent performance to EnsemPro in this evaluation, although results may not be exact due to variations in the dataset (see Methods).

## Conclusions

Profisi Ensemble uses a two layer approach to prediction. Two SVMs are trained using scores from existing prediction programs as features. The predictions from these SVMs are then combined in an either/or manner.

In this work, we have demonstrated the substantial heterogeneity of promoter predictions from current methods. We showed that this heterogeneity enables performance improvements via an ensemble approach. Finally, we have shown that high-sensitivity and high-specificity classifiers may be combined using a "punting" approach to guarantee higher performance across a range of thresholds.

In many fields, diverse predictors for the same task exist, often of broadly similar performance. If these predictors are sufficiently heterogenous, there is merit in exploring an ensemble-based approach. If high specificity/precision is required, consideration should be given to using feature ranking to ensure that only useful features are included.

The same technique we have used for human predictions could be extended to any other genome, as long as sufficiently diverse predictions are available for it. Detailed instructions on applying our method to other organisms are included in Additional file 2. Many prediction programs are able to output predictions for multiple genomes. EP3, for example contains models for ten model organisms [25]. As we have used a supervised approach, a high quality training set (preferably based on experimental data, like the dbTSS) is essential, however.

5 bp resolution probability scores for genome builds hg17 and hg18 are available from http://mlg.ucd.ie/profisiensemble. 1 bp resolution scores are available on request. Source code is available in Additional file 3.

## Methods

To assess the overlap between predictions from different programs, whole genome predictions were downloaded from the UCSC Genome Browser [30] and from the websites associated with the programs. Where multiple predictions existed around a single locus (2000 base pairs), only the prediction with the highest score was kept. Programs giving discrete predictions (N-SCAN [28], FirstEF [26], and Eponine [27]) had roughly 20,000 predictions each. The remaining programs gave continuous scores for the whole genome. These scores were thresholded to also leave ~20,000 predictions per program. Overlap between sets was measured by dividing set intersection by set union for each pair of programs. Overlap was measured for (a) all predictions, (b) true positive predictions only, and (c) false positive predictions only. Predictions within 1,000 bp of the 5' end of a RefSeq first exon were counted as true positives.

N-SCAN, FirstEF, and Eponine predictions were downloaded from the UCSC Genome Browser. These point predictions were converted to continuous scores using a 1000 base pair window, with the central 200 base pairs getting the full score, linearly falling to 0 at the edges, giving a trapezoid-type distribution. These parameters were determined using small-scale tests on the ENCODE regions. The remaining features had scores for all base pairs. ProSOM and EP3 predictions were obtained using the Java executables available online. ARTS predictions were download from the ARTS website. Profisi melting temperatures were downloaded from the human genome melting map. Methylation scores were obtained from a whole-genome methylation map of 15 cell lines [19] (Island methylation scores from Supplementary Table 1b). Cell lines were split into pluripotent and differentiated categories, and averaged. Scores for the two sperm cell lines were ignored due to the large differences in DNA packing and methylation in these lines. PhastCons 17-way vertebrate conservation scores were downloaded from the UCSC Genome Browser.

Training examples were drawn from the 44 ENCODE regions which together comprise about 1% of the human genome. Positive examples were taken from the dbTSS [31], an experimentally verified database which is already used as the training set for [9] and [24]. There were 519 TSS from the database in the ENCODE regions. Five times as many negative examples were selected, to account for the greater variety of negative examples (intergenic, exons, introns, non-promoter regulation such as enhancers, insulators, etc.). These negative examples were all at least 1000 base pairs from the nearest TSS.

Principal components analysis was performed in Weka 3.6 [32] using the default parameters, giving five principal components. Information gain-based feature selection was also performed in Weka using the default parameters.

LibSVM 2.9 [33] was used to train the models and generate predictions, due to its speed, stability, and availability for multiple platforms. It is not multithreaded, but was easily parallelizable as each chromosome was a separate test file. The default kernel - the radial basis function (RBF) was used. Weights were used to compensate for the uneven class sizes. Features were normalized in the range 0-1 to maximize sparsity. LibSVM was set to output a probability rather than a margin score. The error penalty (C) and the tightness parameter (γ) were chosen using the supplied grid.py.

Figure 3 shows performance on the mapped portions of human chromosome 22 (~1% of the genome). The reduced set outperforms the full set above a certain crossover point. The probability from the reduced set at this crossover was 0.94. To ensure that this was not due to the SVM parameters resulting in optimization of different areas of the curve, a wide range of values of C and γ were tried. In all cases, the area under the curve was reduced, but the shape of the curve stayed the same. Based on this, we decided to combine predictions from both with an either/or approach. Reduced model predictions below 0.94 were discarded and replaced with predictions from the full set, which were scaled so that the highest value remaining was 0.94.

As the evaluations for both MetaProm and EnsemPro are based on the older system of point predictions, the continuous scores from Profisi Ensemble had to also be converted to point predictions. We did this using a combination of thresholding and clustering. Thresholding meant throwing away all predictions below a certain level. Clustering meant finding the location with the highest score, and discarding all locations within n base pairs of it, then finding the location with the next highest score and doing the same, etc., until the last location was reached. For both the MetaProm and EnsemPro evaluations, we performed a grid search on the thresholding and clustering parameters, and kept the best performing ones. Cluster sizes were 50-2000 for EnsemPro and 500 for MetaProm. Thresholds were 0-1 for both. Predictions in areas not examined by MetaProm and EnsemPro were discarded.

42,536 TSS in 14,566 sequences were obtained from the MetaProm authors, along with sensitivity-specificity curves. MetaProm CpG and non-CpG scores were combined.

The EnsemPro evaluation describes discarding EPD TSS where there missing bases within 1,150 bp of the TSS, leaving 400 TSS from 1871. As we were unable to find any missing bases, we used all TSS. EnsemPro figures were obtained from Table 2 of the EnsemPro paper. Weighted majority voting figures were used, as this was the best performing method.

Predictions were made for genome build hg17, and reduced to 5 base pair resolution and converted to build hg18 for testing with pppBenchmark.

## Authors' contributions

DH and PC suggested the project and edited the manuscript. DD designed and performed the analysis, created and evaluated the predictions, and drafted the manuscript. MS performed PCA analysis. All authors read and approved the final manuscript.

## Supplementary Material

Additional file 1 **pppBenchmark CAGE evaluation**. Evaluation of Profisi Ensemble versus ARTS, EP3, and ProSOM using pppBenchmark protocol 2A, for all predictions, CpG predictions, and non-CpG predictions. Click here for file

Additional file 2 **Instructions for other organisms**. Instructions for implementing the Profisi Ensemble method for organisms other than human Click here for file

Additional file 3 **Profisi Ensemble source**. Java source code for routines used to preprocess data and process results Click here for file
