# Supplementary material for: Ensemble approach combining multiple methods improves human transcription start site prediction
Source: BMC Genomics. 2010 Nov 30;11:677. doi: 10.1186/1471-2164-11-677 (PMC3053590; doi:10.1186/1471-2164-11-677)
Supplement: Additional file 2 — Instructions for other organisms. Instructions for implementing the Profisi Ensemble method for organisms other than human [file 1471-2164-11-677-S2.DOC]

### Creating your own ensemble predictions

If you have multiple prediction sets (e.g. for organisms other than human, or even for prediction of other features), you can generate a single set of new, more accurate predictions using the same methods used to generate the predictions seen above. The majority of work is done by LibSVM and its associated scripts. We also performed some text file manipulation using our own routines, which we have [made available for download](http://bioinf.ucd.ie/files/pe_src.zip) , but you could easily use your favourite Python libraries, e.g. [PyCogent](http://pycogent.sourceforge.net/), or a tookit such as [Gff Tools](http://biowiki.org/GffTools/). When feature data was based on different assemblies for the same organism, we used [LiftOver](http://genome.ucsc.edu/cgi-bin/hgLiftOver) to standardise on a common assembly. Note that a command line version is also available for large datasets.

1. **Decide on your training set**
   For your training set, you will need a (hopefully) high quality reference annotation. For human promoter predictions, we used the [dbTSS](http://dbtss.hgc.jp/). If an organism has a high quality gene annotation available, the start of the 5' UTR may be assumed to be a TSS. For mouse, the [CAGE database](http://fantom3.gsc.riken.jp/) contains accurate TSS locations, although sites with low numbers of tags may need to be discarded.
2. **Generate negative examples**
   You will need to give LibSVM negative examples so that it can find a decision boundary between positive and negative classes. The simplest method is to sample randomly from the genome, excluding samples that are too close to the positive set you defined in step 1. The definition of 'too close' may differ for different organisms; values of around a thousand should work well for mammals. You will also need to decide how many negative examples you use - we used five times as many negative as positive examples, as the negative class could be thought to be more diffuse (comprising introns, exons, intergenic, etc.).
3. **Get your training data**
   You will need several input features. Features can be predictions from other programs, or anything known to be correlated with promoters. Features will typically be either a number of discrete regions, or a continuous series of scores. You will need to get the values of your features at each of the positions in your training set. The routines we have provided may be of use here, but will need to be modified for your data. LibSVM takes data in sparse format. Use 1 as the class label for a promoter, and -1 for a non-promoter. A line with 4 features:

1 1:0.7875 2:0 3:1.287 4:2

Data should be normalised:

svm-scale -l 0 -u 1 -s training.mults training_set > scaled_set

This scales each feature to between 0 and 1. The use of 0 as the lower bound will help ensure more zero values (greater sparsity), and hence better performance, than using, say, -1.

1. **(Optional) analyse in Weka**
   [Weka](http://www.cs.waikato.ac.nz/ml/weka/) is a machine learning toolkit which may be used for data exploration. You can use Weka to discard features that may not boost the accuracy of your classifier. Under the *Select attributes* tab, choose *WrapperSubsetEval* as your evaluator. You may wish to discard poorly performing features.
2. **Train your LibSVM classifier**
   For the low dimensional numeric data we are using, the default radial basis function is perfect. It has two parameters which need to be chosen using cross-validation. This is done using the grid.py script in the tools directory.

./grid.py -w1 weight

where weight is the ratio of negative to positive examples. Grid.py will call gnuplot to graphically show performance across a range of parameter values. Take note of the best values for C and gamma and then use them to train the SVM:

svm-train -g gamma -c cost -w1 weight -b 1 scaled_set

1. **Get your test data**
   You will need one or more test data files in the same format as the training file. We used a file for each chromosome, with a line for each base pair. The preprocessing required to convert your data can be difficult. It may be useful to 'unroll' complex annotations into a simple list of numbers, one per base pair. Our included code contains a number of routines for this. The test data should be scaled using the same scales as the training data, to ensure we compare like with like.

svm-scale -l 0 -u 1 -r training.mults test_set > scaled_set

1. **Make predictions**
   The -b 1 parameter tells LibSVM to output probabilities for each test case, rather than a simple class label. LibSVM is single-threaded, but obviously if we have multiple test files then we can run multiple copies simultaneously.

svm-predict -b 1 scaled_test model_file output_probs

1. **Create predictions in the format of your choice**
   We now have a probability value for each test case, and can iterate through the file to create a .WIG file suitable for upload to the UCSC Genome Browser, or discard all values below a certain threshold and output the remaining to a .BED or .GFF file.
